# Immune adaptor ADAP in T cells regulates HIV-1 transcription and cell-cell viral spread via different co-receptors

**DOI:** 10.1186/1742-4690-10-101

**Published:** 2013-09-18

**Authors:** Bin Wei, Lei Han, Truus E M Abbink, Elisabetta Groppelli, Daina Lim, Youg Raj Thaker, Wei Gao, Rongrong Zhai, Jianhua Wang, Andrew Lever, Clare Jolly, Hongyan Wang, Christopher E Rudd

**Affiliations:** 1 The State Key Laboratory of Cell Biology, Institute of Biochemistry and Cell Biology, Shanghai Institutes for Biological Sciences, Chinese Academy of Sciences, Shanghai China; 2 Department of Medicine, Level 5, Addenbrooke’s Hospital, Hills Road, Cambridge CB2 2QQ, UK; 3 Division of Infection and Immunity, University College London, London WC1E 6BT, UK; 4 Cell Signalling Section, Division of Immunology, Department of Pathology, Tennis Court Road, University of Cambridge, Cambridge CB2 1QP, UK; 5 Institute Pasteur of Shanghai, Chinese Academy of Science, Shanghai China; 6 Cambridge Institute of Medical Research, Hills Road, CB2 OXY, Cambridge, UK; 7 TEMA current address: Centre for Childhood White Matter Disorders, VU University Medical Centre, De Boelelaan 1087, 1081HV, Amsterdam, the Netherlands

**Keywords:** ADAP, HIV-1 transcription, HIV-1 transmission, Integrin, Virological synapse

## Abstract

**Background:**

Immune cell adaptor protein ADAP (adhesion and degranulation-promoting adaptor protein) mediates aspects of T-cell adhesion and proliferation. Despite this, a connection between ADAP and infection by the HIV-1 (human immunodeficiency virus-1) has not been explored.

**Results:**

In this paper, we show for the first time that ADAP and its binding to SLP-76 (SH2 domain-containing leukocyte protein of 76 kDa) regulate HIV-1 infection via two distinct mechanisms and co-receptors. siRNA down-regulation of ADAP, or expression of a mutant that is defective in associating to its binding partner SLP-76 (termed M12), inhibited the propagation of HIV-1 in T-cell lines and primary human T-cells. In one step, ADAP and its binding to SLP-76 were needed for the activation of NF-κB and its transcription of the HIV-1 long terminal repeat (LTR) in cooperation with ligation of co-receptor CD28, but not LFA-1. In a second step, the ADAP-SLP-76 module cooperated with LFA-1 to regulate conjugate formation between T-cells and dendritic cells or other T-cells as well as the development of the virological synapse (VS) and viral spread between immune cells.

**Conclusions:**

These findings indicate that ADAP regulates two steps of HIV-1 infection cooperatively with two distinct receptors, and as such, serves as a new potential target in the blockade of HIV-1 infection.

## Background

Infection with the human immunodeficiency virus-1 (HIV-1) causes a severe and selective depletion of CD4^+^ T lymphocytes in the immune system [1,2]. HIV-1 binds primarily to CD4 together with chemokine receptors CXCR4 or CCR5. Receptor engagement induces a conformational change in the HIV envelope glycoprotein (Env), which mediates membrane fusion and viral penetration. Replication of HIV-1 is mediated primarily by transcription factors such as NFAT, AP1 and NF-κB [3,4]. NF-κB regulates long terminal repeat (LTR) activation within the HIV-1 genome by interacting with tandem binding sites in the enhancer region and mutant IκB alpha inhibits *de novo* HIV-1 infection in T cells [5-7]. Mutations within internal TATA sequences or the NF-κB binding sites also impair LTR activity and viral replication [8].

HIV-1 can disseminate between immune cells either by cell-free infection or by direct cell-cell spread. Cell-cell transmission of HIV-1 takes place through membrane nanotubes or virological synapses (VS) that form following physical contact between infected and uninfected cells [9-13]. Electron micrographs have shown HIV-1 accumulation at the interface between HIV-1 infected and uninfected cells [11,14], while immunofluorescence microscopy and time-lapse imaging have shown the accumulation of viral proteins at the contact interface as well as the movement of viruses from one cell to another [11,15-17]. This mode of dissemination is at least 500-fold more efficient than infection by cell-free virus [10,16,17], which may facilitate HIV-1 spread within secondary lymphoid tissues [18]. Further, infected dendritic cells (DCs) and macrophages use the VS to transfer HIV-1 to T cells [19,20]. Spread via synapses requires the localization of CD4, CXCR4 or CCR5 as well as the integrin lymphocyte function-associate antigen 1 (LFA-1) and intercellular adhesion molecule-1 (ICAM-1) at the site of cell-cell contact [10-13,17,20]. The blockade of LFA-1 reduces VS formation [12], and more importantly, DCs isolated from leukocyte adhesion deficiency (LAD)-I patients show decreased viral spreading to CD4^+^ T-cells [21]. Furthermore, LFA-1 and ICAM-1 from host cells can be incorporated into HIV particles for enhanced infectivity [22,23].

The activation status of T-cells plays an important role in facilitating viral replication and spread since HIV-1 replicates inefficiently in quiescent T cells [24]. In this context, immune cell specific adaptor proteins that mediate T-cell activation and effector functions have been identified [25,26]. These adaptors lack definable catalytic activities, but instead, possess binding domains or sites for the formation of multimeric complexes. Of these, Linker of activated T cells (LAT) and Src homology 2 (SH2) domain-containing leukocyte protein of 76 kDa (SLP-76) (also named *lcp2,* lymphocyte cytosolic protein 2) are needed for antigen-receptor induced calcium mobilization [27,28]. SLP-76 binds to ADAP (adhesion- and degranulation-promoting adaptor protein, also named as Fyb [fyn binding protein] or SLAP-130 [SLP-76-associated phosphoprotein of 130 kDa]), which is needed for up-regulation of LFA-1 adhesion [29-31]. This pathway is mediated downstream by SKAP1 (*Src* kinase-associated phosphoprotein 1) that regulates the complex formation between Rap1 and RapL (regulator for cell adhesion and polarization enriched in lymphoid tissues) [26,32-36]. Two tyrosine motifs at Y^595^DDV and Y^651^DDV of ADAP bind to the SH2 domain of SLP-76 upon TCR stimulation. A double point mutation in ADAP at Y^595^F and Y^651^F (termed M12) is defective in SLP-76 binding and shows reduced LFA-1 adhesion and pSMAC formation [31,34]. Despite this, a potential connection between ADAP and HIV-1 infection has not been explored.

In this study, we demonstrate that ADAP and its binding to SLP-76 regulate two steps of HIV-1 infection by cooperating differentially with two distinct co-receptors. Loss of ADAP and the SLP-76/ADAP module markedly impaired CD28-mediated HIV-1 transcription as well as LFA-1-dependent formation of virological synapse for cell-cell viral spread. These findings identify ADAP and its signaling module as key regulators of HIV-1 infection.

## Results

### Disruption the SLP-76-ADAP signaling module inhibits HIV-1 infection

**Figure 1:**
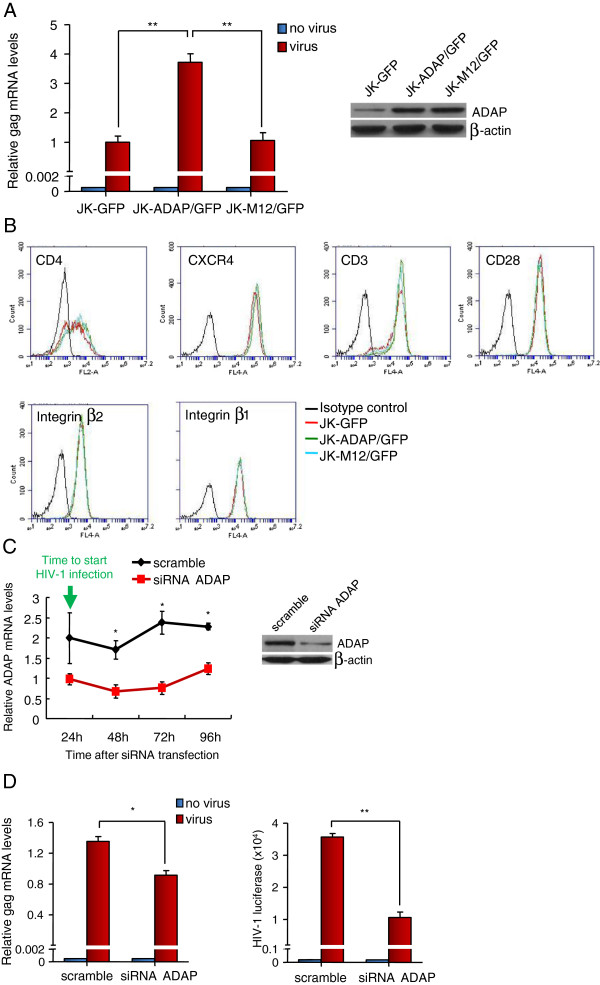
**Disruption the SLP-76-ADAP signaling module inhibits HIV-1 infection in T-cells. (A)** Jurkat T cells were stably transfected with GFP, ADAP/GFP or M12/GFP and infected with single-cycle luciferase reporter HIV-1 (equivalent to 5 ng p24^Gag^/10^6^ cells). The HIV-1 gag mRNA levels were determined by qRT-PCR 72 hrs post infection (P = 0.032, left and P = 0.005, right). The ADAP/M12 expression levels were examined by immunoblotting. **(B)** Overexpression of ADAP/GFP or M12/GFP in Jurkat cells did not alter the surface expression levels of CD4, CXCR4, CD3, CD28, β1 or β2 integrins as determined by flow cytometry. **(C)** Human primary CD4^+^ T cells were transfected with the scramble siRNA or siRNA targeting ADAP, and the knockdown efficiency was confirmed by immunoblotting (48 hrs post-transfection) or by qRT-PCR at different time points during HIV-1 infection. **(D)** Human primary CD4^+^ T cells were transfected with siRNAs for 24 hrs, then infected with single-cycle luciferase reporter HIV-1. The HIV-1 gag mRNA levels were determined by qRT-PCR 72 hrs post infection (left panel, P = 0.0192). Alternatively, the amount of HIV-1 was measured according to the luciferase readings (right panel, P = 0.0032) (* represents p = <0.05, ** represents p = <0.01).

We and others have previously outlined the importance of the SLP-76-ADAP-SKAP1 pathway in the activation of LFA-1 [31-36]. A mutant of ADAP lacking tyrosine residues 595 and 651 (termed M12) is unable to bind to SLP-76 and impairs LFA-1 activation [31,34]. We assessed whether wild-type ADAP and the mutant M12 could regulate HIV-1 infection in Jurkat T-cells (Figure 1A,B). Jurkat T-cells were stably transduced with retroviral supernatants encoding ADAP-IRES-GFP or M12-IRES-GFP (termed JK-ADAP/GFP and JK-M12/GFP) or with GFP alone (JK-GFP). Expression remained stable due to integration. The transfectants showed the same expression levels of CD4, CXCR4, CD3, CD28, β1 and β2 integrins as the control GFP expressing Jurkat cells as measured by flow cytometry (Figure 1B). We next infected these cells with a single-cycle HIV-1 virus carrying a luciferase reporter (equivalent to 5 ng p24^Gag^). The mRNA levels of HIV-1 gag were measured at 72 hours post-infection by quantitative RT-PCR (qRT-PCR) with specific primers for HIV-1 gag. JK-ADAP/GFP cells showed 3-4-fold higher levels of HIV-1 gag mRNA when compared to JK-GFP cells. By contrast, JK-M12/GFP cells failed to support the increase of HIV-1 gag mRNA beyond that observed in the JK-GFP cells (Figure 1A). The level of transfected M12 was similar to ADAP as seen by western blotting (Figure 1A, upper right inset). We confirmed that after HIV-1 infection, overexpression of ADAP/GFP or M12/GFP had no effects on CD4 or CXCR4 expression in Jurkat cells (Additional file 1: Figure S1A).

We next stably overexpressed GFP, ADAP or M12 into human C8166 T cells (C8166-GFP, C8166-ADAP and C8166-M12) (Additional file 1: Figure S1B). These cells were infected with low dose or high dose of HIV-1 (equivalent to 1.5 or 15 ng p24^Gag^, respectively). Supernatants were collected and quantified by ELISA for levels of of HIV-1 p24^Gag^ at various times post-infection. We found that at both doses of input virus, C8166-M12 cells were impaired in their support of HIV-1 replication relative to cells expressing wild-type ADAP. When we used low dose of virus to infect cells, C8166-ADAP cells and the control cells supported productive infection, whereas C8166-M12 cells failed to produce the detectable levels of p24^Gag^ (Additional file 1: Figure S1B, right panel). Over 95% of C8166 T cells overexpressed GFP, or ADAP/GFP or M12/GFP (Additional file 1: Figure S1B, left and middle panels), which had no effect on the expression of surface receptors (Additional file 1: Figure S1C) and showed similar growth rates (Additional file 1: Figure S1D). We further examined whether HIV-1 infection of human primary CD4^+^ T cells was dependent on ADAP (Figure 1C,D). ADAP expression was reduced using specific siRNAs. qRT-PCR showed a 50-60% reduction in ADAP mRNA transcripts over a period of 96 hours post-transfection (Figure 1C). Similarly, western blotting of cells at 48 hours confirmed the significantly reduced ADAP expression after transfection with siRNA-ADAP (Figure 1C, right inset). siRNA transfected human CD4^+^ T cells were then infected with the single-cycle HIV-1 virus containing luciferase reporter [12]. siRNA for ADAP reduced HIV-1 gag mRNA levels by 30% when assessed at 72 hours post-infection (Figure 1D, left panel). A measurement of luciferase activity confirmed that siRNA for ADAP resulted in a significant reduction of HIV-1 infection (Figure 1D, right panel). The surface expression of CD3, CD4, CD28, CXCR4, β1/β2 integrins and ICAM-1 in human CD4^+^ T cells was not affected by knockdown of ADAP (Additional file 1: Figure S1E). Collectively, these data indicate that ADAP is needed for the optimal HIV-1 infection of T-cell lines and primary human T-cells.

### ADAP and SLP-76 regulates HIV-1 LTR transcription in a CD28- and NF-κB-dependent manner

**Figure 2:**
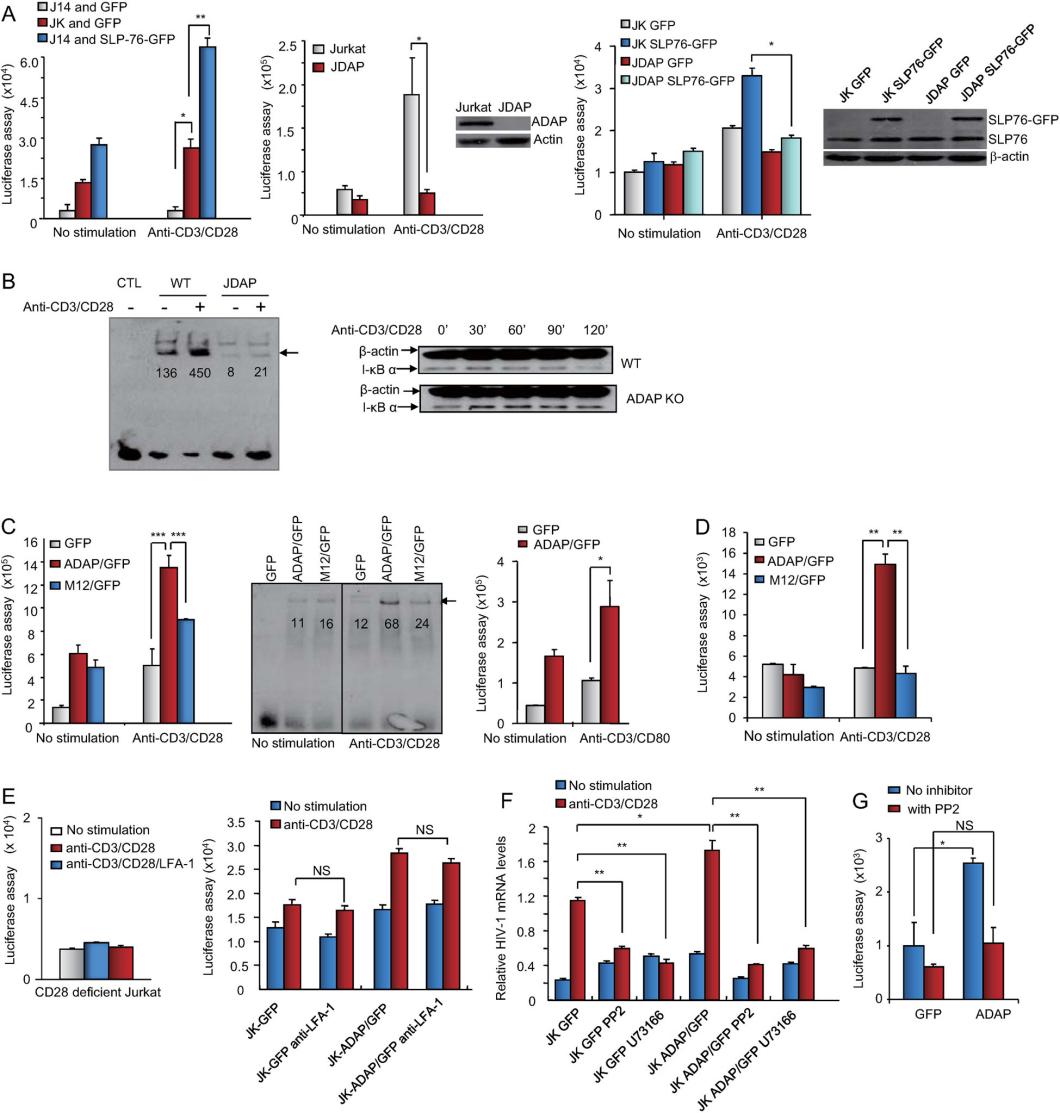
**ADAP and SLP-76 regulates HIV-1 LTR transcription in a CD28- and NF-κB-dependent manner. (A)** The pLTR-gag3-flag-luc luciferase reporter plasmid was transfected together with GFP or SLP-76-GFP into Jurkat, SLP-76 deficient J14 cells (upper left panels, P = 0.0112 left; P = 0.002 right) or ADAP deficient JDAP cells (lower left panel, P = 0.0145). Alternatively, this reporter plasmid was transfected into Jurkat or JDAP cells (upper right panel, P = 0.0203). These cells were stimulated with or without anti-CD3/CD28 to measure HIV-1 LTR transcription. **(B)** EMSA assay and immunoblotting with anti-IκBα showed that the loss of ADAP markedly decreased anti-CD3/CD28-induced NF-κB activation. The absolute integrated OD values of each band were analyzed by Gel-Pro (left panel). **(C)** Plasmids expressing GFP or ADAP/GFP or M12/GFP were transfected together with the luciferase reporter plasmid into Jurkat cells to assess the luciferase activity after anti-CD3/CD28 (left panel, P < 0.0001) or anti-CD3/CD80-Fc (right panel, P < 0.05) stimulation. The NF-κB activation was examined by an EMSA assay (middle panel). **(D)** Human primary PBLs were transfected with GFP or ADAP/GFP or M12/GFP together with the luciferase report plasmid to measure HIV-1 LTR transcription (P < 0.01). **(E)** CD28 deficient Jurkat cells (upper panel) or wild type Jurkat cells (lower panel) were stimulated by anti-CD3/CD28 with or without anti-LFA-1 blocking to check HIV-1 transcription (P > 0.05). **(F)** Jurkat cells expressing GFP or ADAP/GFP were treated with indicated specific inhibitors, followed by a measurement of the HIV-1 mRNA levels. **(G)** Human primary PBLs were transfected with GFP or ADAP/GFP, stimulated by anti-CD3/CD28 with or without the src kinase inhibitor PP2 to measure HIV-1 LTR transcription (NS/not significant; P = 0.1465). Three independent experiments were performed and the representative data are shown with error bars (*p = <0.05, **p = <0.01).

To uncover the molecular basis of ADAP involvement in HIV-1 infection, we firstly examined its potential effects on the induction of HIV-1 LTR transcription. Wild type, SLP-76-deficient Jurkat T-cells (termed J14) or ADAP-deficient Jurkat T-cells (termed JDAP) [37] were transfected with a pLTR-gag3-flag-luc reporter plasmid followed by stimulation via anti-CD3/CD28 ligation for 6 hours. The pLTR-gag3-flag-luc plasmid contains the HIV-1 5’ LTR promoter region with two NF-κB binding sites and a firefly luciferase open reading frame [38]. HIV-1 transcription was then assessed by a measure of luciferase activity (Figure 2A). Anti-CD3/CD28 stimulation induced a two-fold increase in HIV-1 transcription in wild type Jurkat cells, an effect that was not seen in J14 cells (Figure 2A, upper left panel). Re-expression of SLP-76 into J14 cells restored and enhanced HIV-1 transcription (blue bars). Similarly, anti-CD3/CD28 induced HIV-1 transcription was markedly impaired in ADAP-deficient JDAP cells (Figure 2A, upper right panel). Furthermore, the overexpression of SLP-76 in JDAP cells did not bypass or compensate for the ADAP deficiency (Figure 2A, lower panel). Anti-CD3/CD28 increased NF-κB binding by over 3-fold in wild type Jurkat cells as measured by an electrophoretic mobility shift assay (EMSA) (the integrated OD values from Gel-Pro image analysis software: 136 to 450), but not in JDAP cells (Figure 2B, left panel). In agreement with this, a difference in the degradation of IκB alpha was observed between wild type and JDAP cells (Figure 2B, right panel). IκB alpha is an inhibitor of NF-κB which is degraded in response to TCR/CD28 ligation [39]. Anti-CD3/CD28 induced a degradation of IκB alpha in Jurkat cells over a period of 90-120 min, an effect that was not observed in JDAP cells. Instead, JDAP cells sustained IκB alpha expression over the time course. These observations indicate that ADAP expression is needed for anti-CD3/CD28 induced NF-κB activation.

We also assessed the effect of ADAP binding to SLP-76 to regulate HIV-1 transcription by using cells transfected with wild type ADAP or the mutant M12 (Figure 2C). ADAP expression increased anti-CD3/CD28 induced transcription of the HIV-1 LTR by 2.5 fold, while this was impaired with M12 (i.e. 30-40 percent less than observed for ADAP, Figure 2C, left panel). ADAP overexpression also increased HIV-1 transcription in response to anti-CD3 and CD80-Fc, the natural ligand for CD28 (Figure 2C, right panel). Further, an EMSA assay showed that ADAP increased NF-κB activation (OD values from 11 to 68), and this increase was blocked by M12 (OD values from 16 to 24) (Figure 2C, middle panel). As a control, ADAP could not further enhance the activity of a HIV-luciferase reporter lacking NF-κB binding sites (Additional file 2: Figure S2A). Significantly, the same inhibitory effect of M12 was noted in primary human T-cells that had been co-transfected with pLTR-gag3-flag-luc and ligated with anti-CD3/CD28. ADAP expression increased HIV transcription by 2.5-3 fold, whereas M12 had no effect (Figure 2D). These data indicate that ADAP and its binding to SLP-76 cooperate with CD28 co-ligation to regulate LTR activity in Jurkat and human primary T-cells.

Stimulation of T-cells from CD28 deficient Jurkat cells further showed a dependency of the NF-κB-driven HIV-1 transcriptional response on CD28 (Figure 2E, left panel). Anti-LFA-1 antibody (i.e. anti-CD18) ligation had no ability to activate HIV-1 transcription alone, or in conjunction with CD3/CD28 ligation or ADAP/GFP expression (right panel). These data indicate that CD28, but not LFA-1 costimulation, cooperates with ADAP in the activation of HIV-1 transcription.

We next determined whether ADAP activation of the HIV-1 LTR intersects with other signaling events. Specific inhibitors of src kinases, phosphotidylinositol 3 kinase (PI 3 K) and phospholipase C (PLC) were used in conjunction with anti-CD3/CD28 stimulation. We measured the HIV-1 gag mRNA levels by qRT-PCR (Figure 2F) or HIV 5’ LTR transcription activity (Additional file 2: Figure S2B). Src kinase inhibitor PP2 and PLC inhibitor U73122 significantly decreased anti-CD3/CD28 induced HIV-1 transcription in Jurkat cells, and reduced the increase observed in ADAP/GFP expressing cells. The inhibitory effect of PP2 on HIV-1 transcription was also observed in primary human T-cells (Figure 2G), showing that ADAP expression increased anti-CD3/CD28 induced transcription by 2.5-fold, which was blocked by PP2 treatment. The PI 3 K inhibitor LY294002 however did not affect transcription (Additional file 2: Figure S2B). We previously showed that this concentration of LY294002 effectively inhibited PI 3 K in Jurkat cells by examining protein kinase B (AKT/PKB) phosphorylation [40]. These data indicate that src kinases and PLC are needed for ADAP enhancement of anti-CD3/CD28 induced HIV-1 transcription.

### LFA-1 dependency in ADAP-induced HIV-1 infection

**Figure 3:**
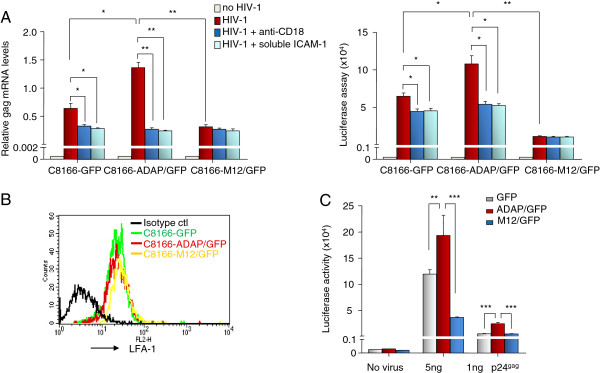
**LFA-1 dependency in ADAP-induced HIV-1 infection. (A)** Single-cycle luciferase reporter HIV-1 was incubated for 2 hrs at 37 degree with C8166 cells that over-expressed GFP control or ADAP/GFP or M12/GFP, in the absence or presence of soluble ICAM-1-Fc or anti-CD18 treatment. After washing, these C8166 cells were further cultured for 72 hrs with or without soluble ICAM-1-Fc or anti-CD18. The amount of HIV-1 was measured by qRT-PCR using primers specific for the HIV-1 gag mRNA relative to an actin housekeeping gene (left panel) or was determined by luciferase assay (right panel). **(B)** Overexpression of ADAP or M12 in C8166 cells did not alter the surface expression levels of LFA-1. **(C)** Low (1 ng p24^Gag^) or high (5 ng p24^Gag^) amount of single-cycle luciferase reporter HIV-1 viruses were used to infect C8166 cells over-expressing GFP control or ADAP/GFP or M12/GFP. The luciferase readings were collected from triplicate samples with error bars (* represent p = <0.05, ** represents p = <0.01, *** represents p = <0.001).

The striking effects of M12 on HIV-1 replication suggested that additional mechanisms might also be operating. Of particular interest was the involvement of ADAP on the activation of LFA-1 for adhesion [31,34]. To assess the dependency on ADAP-induced LFA-1 adhesion during HIV-1 infection, single-cycle HIV-1 containing a luciferase reporter was incubated with C8166 cells expressing GFP control, ADAP/GFP or M12/GFP. These cells were either left untreated, or incubated with virus in the absence or presence of soluble ICAM-1-Fc or anti-LFA-1 to block LFA-1-mediated adhesion (Figure 3A). HIV-1 gag mRNA levels were assessed using qRT-PCR with specific primers for HIV-1 gag (Figure 3A, left panel). Gag mRNA levels were reduced in the control/GFP (i.e. from 0.7 to 0.3, p ≤ 0.05) and ADAP/GFP cells (i.e. from 1.4 to 0.3, p ≤ 0.01) in the presence of soluble ICAM-1-Fc treatment. In contrast, the already impaired levels of HIV-1 gag mRNA production in M12/GFP cells (i.e. 1.4 vs. 0.35 when compared to ADAP/GFP cells, p ≤ 0.01) was not further reduced by the presence of ICAM-1-Fc. Similar to the blocking effect by soluble ICAM-1-Fc, anti-LFA-1 antibody also impaired HIV-1 gag mRNA production (Figure 3A, left panel). We also measured the luciferase reading from the single-cycle luciferase reporter HIV-1-infected cells, where soluble ICAM-1-Fc or anti-LFA-1 treatment reduced luciferase readings in the controls and ADAP/GFP expressing C8166 cells (Figure 3A, right panel). Consistently, the decreased luciferase reading in M12/GFP cells was not further reduced by soluble ICAM-1-Fc or anti-LFA-1 treatment (Figure 3A, right panel). As a control, flow cytometry confirmed that ADAP and M12 did not alter LFA-1 expression (Figure 3B). In addition, using higher levels of HIV-1 particles (5 ng p24^Gag^), ADAP expression enhanced the luciferase values relative to the GFP transfected control, while M12 markedly decreased the amount of viruses). Consistent results were also observed when these cells were incubated with lower quantities of virus (1 ng p24^Gag^) (Figure 3C). These data indicate that the SLP-76-ADAP module regulates HIV-1 infection in an LFA-1-dependent manner.

### SLP-76-ADAP regulates the VS formation between T-cells and DCs

**Figure 4:**
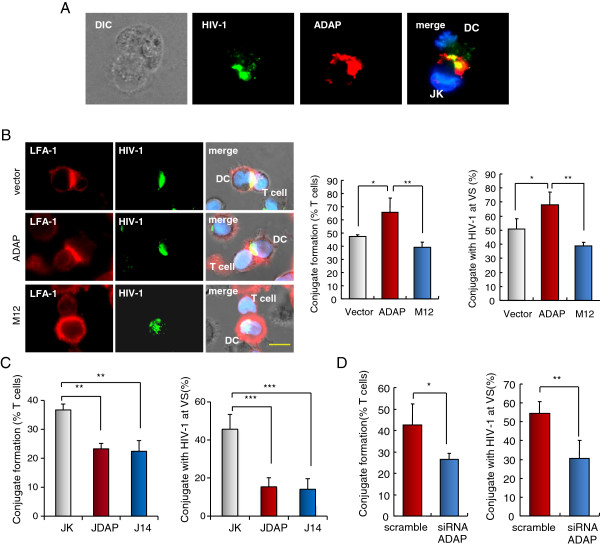
**SLP-76-ADAP regulates the VS formation between T-cells and HIV-1-pulsed DCs.** Jurkat, JDAP or J14 cells were incubated for 30 min with mature human DCs that were pre-pulsed with HIV1-gag-GFP VLPs. Conjugate formation and the presence of HIV1-gag-GFP at the VS interface was assessed. **(A)** Example of a conjugate shows that anti-ADAP staining in T-cells and HIV1-gag-GFP VLPs in DCs were peaked at the interface between DC and T cell. **(B)** Jurkat cells stably overexpressing GFP, ADAP/GFP or M12/GFP were incubated for 30 min with HIV1-gag-GFP-pulsed human DCs. Represent examples are shown in right panels. Histograms show the percent of conjugate formation (left panel; P = 0.0198 left, P = 0.0068 right) and colocalization of HIV1-gag-GFP and LFA-1 at the VS (right panel; P = 0.0107 left, P = 0.0022 right). **(C)** Deficiency of ADAP (JDAP) or deficiency of SLP-76 (J14) impaired HIV1-gag-GFP and LFA-1 localization at the interface between Jurkat cells and DCs. Histograms show the percent of conjugate formation (upper panel, P = 0.0011 left, P = 0.043 right) or colocalization of HIV1-gag-GFP with LFA-1 at the VS (lower panel, P = 0.0006). **(D)** Human primary CD4^+^ T cells were transfected with the scramble siRNA or siRNA ADAP for 48 Hrs, which were then incubated for 30 min with mature human DCs pre-pulsed with HIV1-gag-GFP VLPs. The conjugate formation (P = 0.0241) and HIV1-gag-GFP at the VS interface (P = 0.0018) were analyzed and showed in histograms. Typical examples were shown in right panel. Typical examples are shown in right panel (* represents p = <0.05, ** represents p = <0.01, *** represents p = <0.001).

The blockade of ADAP-induced HIV-1 infection with soluble ICAM-1-Fc suggested that ADAP might contribute to integrin-mediated viral transmission via affecting the formation of VS and cell-cell conjugation. LFA-1 adhesion is known to modulate T-cell conjugation with dendritic cells (DCs) or T-cells, even in an antigen independent manner; and T-cells or DCs expressing HIV-1 can spread the virus by forming conjugates with non-infected T-cells [11-16]. We therefore investigated whether ADAP and SLP-76 could regulate conjugation between DCs and non-infected T cells. For this, Jurkat, JDAP and J14 cells were incubated for 1 hour with mature human DCs that were pre-pulsed with HIV-1-gag-GFP, followed by the scoring of closely opposed pairs of HIV1-gag-GFP expressing DCs and T-cells. Examples of HIV-1-gag-GFP-pulsed DCs and anti-ADAP stained T-cells are shown (Figure 4A). HIV-1-gag-GFP in DCs and ADAP in T-cells localized at the contact region between cells. Our previous reports have demonstrated that M12 disrupted its binding with SLP-76, and reduced LFA-1-mediated conjugates formation between antigen presenting cells and T cells [31,34]. We asked whether the mutant M12 could impair DC-T conjugation and virological synapse formation. Jurkat T cells stably overexpressing GFP, ADAP/GFP or M12/GFP were incubated with mature human DCs that were pre-pulsed with HIV-1-gag-GFP (Figure 4B). ADAP expression enhanced conjugate formation between DC and T cells (64% vs. 47% compared to vector controls, p ≤ 0.05). By contrast, M12 expression significantly decreased conjugation (64% vs. 38%, p ≤ 0.01). Notably, ADAP also enhanced the formation of VS as defined by the presence of HIV-1 at the interface (65% vs. 49%, p ≤ 0.05), while M12 expression reduced VS formation to 39% (p ≤ 0.01). In the case of vector and ADAP transfected cells, LFA-1 and HIV-1 co-localized at the interface (Figure 4B, right panels).

Further, similar results were seen in comparing conjugation of Jurkat relative to JDAP cells (Figure 4C). JDAP cells formed significantly fewer conjugates than Jurkat cells (i.e. from 37% to 22%; p ≤ 0.01). J14 also blocked the conjugate formation with infected DCs to a similar degree (Figure 4C). Further, amongst the cells that formed conjugates, HIV-1-gag-GFP localization was reduced at the contact region when DCs contacted with JDAP cells or J14 cells (Figure 4C). While 46% of wild-type T-cells showed HIV-1-gag-GFP localization at the VS, only 15% of the JDAP or J14 cells showed this feature (Figure 4C). As noted, deficiency of ADAP in JDAP cells (Figure 1B) or deficiency of SLP-76 in J14 cells (Additional file 3: Figure S3A) did not affect LFA-1 expression. Next, we confirmed that knockdown of ADAP in human primary CD4^+^ T cells decreased both conjugate formation (from 41% to 27%, p ≤ 0.05) and VS formation (from 53% to 31%, p ≤ 0.01) (Figure 4D). Taken together, these data indicate that the loss of ADAP or SLP-76 or disruption of the binding between SLP-76 and ADAP impairs conjugation and VS formation between T-cells and DCs.

### The SLP-76-ADAP module regulates viral transfer between T-cells

**Figure 5:**
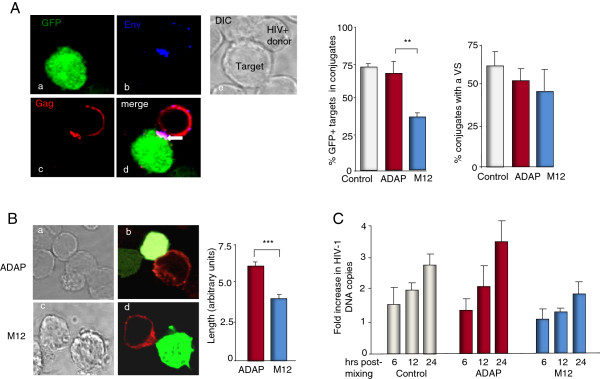
**The SLP-76-ADAP module regulates viral transfer between T-cells. (A)** ADAP/GFP and M12/GFP expressing target cells were mixed with HIV infected donor T cells. Conjugates were defined as closely apposed pairs consisting of one GFP + target cell and a HIV Gag + donor cell. Represent examples show interfacial localization of Gag (red) and Env (blue) proteins between GFP + target cell and a HIV-donor Jurkat cell. Over 200 conjugates from each sample were counted. Data are from three independent experiments and error bars show the SEM (p < 0.05). **(B)** Representative images of ADAP/GFP cell (a,b) and M12/GFP (c,d) cell in contact with a Gag + cell (left panels). Analysis of cell-cell contact surface. Reduced interface size amongst cells that formed conjugates with M12 (p < 0.0001). Bars are represented as means ± SD (n = 32) (right panel). **(C)** M12 reduces HIV-1 spread amongst T-cells. Quantitative RT-PCR was performed using primers specific for the HIV *pol* gene and an albumin house-keeping gene. The ratio of HIV *pol* DNA to albumin was determined as the HIV DNA copy number. The fold increase was calculated relative to the amount of DNA at the time point 0 h. Data are from three independent experiments and error bars show the SEM.

To assess viral-infected T-cells conjugation with non-infected T-cells, ADAP or M12 expressing target T cells (i.e. GFP positive) were incubated with HIV^+^ donor T cells infected with a CXCR4-tropic HIV-1 virus isolate (pNL43). Conjugates were allowed to form for 1 hour with a human non-inhibitory anti-HIV Env monoclonal antibody to stain surface Env (blue), while HIV-1 Gag p17 and p24 were intracellular stained (red) (Figure 5A). Conjugates were scored as apposed pairs consisting of one GFP^+^ target cell and a HIV Gag^+^ donor cell. Examples of a conjugate with the GFP labeled T-cell (panel a), Env staining (panel b), anti-Gag staining (panel c) and the merged image (panel d) are shown (Figure 5A, left panels). While ADAP supported the formation of conjugates amongst 65% of the cells, M12 reduced this to 38% (p = 0.02) (Figure 5A, middle panel). Amongst the remaining cells that formed conjugates, a similar number of ADAP or M12 expressing cells formed a detectable VS region (Figure 5A, right panel). However, a significant difference was noted in the size of the VS interface formed between viral-infected T cells with ADAP or M12 expressing target cells (Figure 5B, p = <0.001). While ADAP expressing cells showed an average contact site of 6.4 units, M12 conjugates reduced this to 4.1 units (Figure 5B). In agreement with this, the loss of ADAP dramatically inhibited anti-CD3 and ICAM-1- induced cell spreading (Additional file 3: Figure S3B).

To assess the effect of ADAP and M12 on HIV-1 transmission between T cells, we next quantified cell-cell spread using a well-defined qRT-PCR assay in which the copy number of HIV-1 *pol* gene was measured and enumerated relative to an albumin housekeeping gene (Figure 5C) [10,13,14]. The fold increase was calculated relative to the number of DNA copies at time point 0 hour to account for the presence of integrated proviral DNA within the infected donor cell population. HIV *pol* copy number corresponds to *de novo* HIV-1 DNA synthesis. An increase above 1 reflects reverse transcription as a result of cell-to-cell spread and new infection of the target cells. While the presence of ADAP sustained cell-to-cell spread, M12 expression induced a significant reduction in viral transfer between cells (i.e. 3.5 vs. 2.2). Overall, these data indicate that M12 effectively reduces the number of T-T cell conjugates and the size of the VS, leading to reduced HIV-1 viral transmission.

## Discussion

Although ADAP acts as an important mediator of T-cell signaling and function [29-32,34,37,41], its role in HIV-1 infection of T-cells had yet to be explored. In this study, we showed that ADAP was a potent regulator of two central events needed for HIV-1 infection, namely, the HIV-1 LTR transcription and viral transfer at the synapses of T-T or DC-T conjugates. Further, the two functions were regulated by two different co-receptors, CD28 in the case of HIV-1 transcription, and LFA-1 in the case of cell-cell transmission. Expression of M12 or the down-regulation of ADAP by siRNA effectively suppressed the propagation of HIV-1. Our findings therefore identify ADAP and the SLP-76/ADAP signaling module as new potential targets for the repression of HIV-1 infection.

Our studies have demonstrated that ADAP regulates two distinct events during HIV-1 infection of T-cells. While NF-κB drives the replication of the long terminal repeat (LTR) [5], the identity of the full range of upstream regulators of NF-κB-LTR is unknown. A variety of pro-inflammatory stimuli such as TNF-α and IL-1 as well as viral proteins and stress inducers are potent activators [42]. In T-cells, protein kinase Cθ (PKCθ) and PKCα activate NF-κB following CD3/CD28 ligation [43-45]. Phorbol ester activation of PKCs can reactivate HIV-1 in cell lines and importantly, in primary quiescent T cells [46,47]. More recently, members of the LAT signalosome including ADAP have been found to be needed for optimal NF-κB activation [41,48]. However, given the different members of the NF-κB family that can be affected by upstream mediators, it has been unclear whether ADAP is needed for HIV-1 LTR transcription. Our findings showed a significant loss of anti-CD3/CD28 induced HIV-1 transcripts in JDAP cells, indicating that ADAP is needed for LTR activation. This in turn was reflected by a lack of detectable IκBα degradation in ADAP deficient JDAP cells. This regulatory event was linked further upstream to SLP-76, since a loss of binding to SLP-76 by the M12 mutant impaired LTR activity in Jurkat and primary human T-cells. It is important to note that overexpression of SLP-76 into JDAP cells did not rescue the defective HIV-1 LTR transcription. This observation suggests that ADAP is the downstream effector of SLP-76 to regulate HIV-1 transcription. Overexpression of SLP-76 increased HIV-1 LTR transcription in WT and SLP-76 deficient J14 Jurkat cells. This effect of SLP-76 on transcription differs from a previous study [49]. The basis of this difference is unclear; however, different results might be caused by different methods used in these studies. Those authors examined the amount of full-length or sliced HIV transcripts by qRT-PCR after J14 or wild type cells were infected with HIV-1 IIIB virus. We used anti-CD3/CD28 to activate J14 or wild type cells and the readout was based on the HIV LTR luciferase reporter assay. The dependency of NF-κB activation on CD28 expression and its engagement in our studies might explain the differences in results. In either case, our findings are consistent with a scenario of SLP-76 upstream regulation of ADAP that in turn is the effector in the regulation of NF-κB transcription.

Further, we observed that the inhibition of Src kinase and PLCγ1 activity blocked ADAP potentiation of HIV-1 LTR transcription in response to anti-CD3/CD28 stimulation. This finding is consistent with the observation that p59^fyn^ can bind and phosphorylate ADAP, while p56^lck^ is potentially involved in NF-κB activation [50]. Consistent with other reports, PLCγ1 activity is required in guanine nucleotide exchange factor Vav-1 induced activation of NF-κB [51]. Overall, our data indicate for the first time that ADAP and SLP-76 are needed for anti-CD3/CD28-induced NF-κB binding to the HIV-1 LTR and optimal HIV-1 transcription.

Our second major observation was that ADAP regulated HIV-1 transmission between DC-T or T-T cells. Evidence has accumulated over the years showing efficient viral spread by direct cell-cell contact [52]. In our study, while the blocking of LFA-1 had no effect on the NF-κB-driven HIV-1 LTR transcription, it nevertheless effectively impaired HIV-1 infection. This observation underscored the distinct nature of the two steps affected by ADAP. JDAP cells and human primary CD4^+^ T cells with reduced ADAP expression by siRNA formed markedly reduced numbers of T-DC conjugates and showed decreased HIV-1-GFP VLP localization at the VS interface. We observed that the M12 mutant also inhibited T-T conjugate formation, while the remaining conjugates showed a reduced size of the interface at VS. Both events would be expected to interfere with the optimal viral spread between cells. Finally, in agreement, the *de novo* HIV DNA synthesis as measured by levels of HIV *pol* in T-cell cultures confirmed a significant reduction in viral spread.

The identity of other signaling mediators other than *src* kinases and phospholipase C that cooperate with ADAP to regulate the VS formation and cell-to-cell viral spread remains to be determined. ITK and ZAP-70 are needed for viral cell-cell transmission [53,54], whereas ADAP has additional binding sites for vasodilator-stimulated phosphoprotein (VASP), a regulator of actin branching [55]. LFA-1 ligation can re-model actin in T-cells [31,56,57] and T cells require actin polymerization for HIV-1polarization at the cell-cell contact area. This in turn is needed for the proper formation of the VS between T-cells, as well as the efficient entry of HIV-1 into activated CD4^+^ T cells [57]. In agreement, we observed reduced cell spreading in JDAP cells, as well as a reduced interface between HIV-1 infected T cells and non-infected M12 cells. The inside-out pathway is linked ADAP with the downstream SKAP-1, which is needed for the RapL-Rap1 complex formation and binding of this complex to the cytoplasmic tail of LFA-1 [32,33,35,36,58]. In this context, LFA-1 also determines the preferential infection of memory CD4^+^ T cells by HIV-1 [59]. Together, ADAP and the SLP-76-ADAP complex represent exciting novel targets for reducing two steps of HIV-1 infection.

## Conclusion

This study is the first reported demonstration that ADAP and the SLP-76/ADAP signaling module play central roles in two distinct phases of HIV-1 infection. Firstly, ADAP cooperated with the co-receptor CD28 and TCR to enhance HIV-1 LTR transcription via the regulation of NF-κB. This regulatory event was dependent on expression of co-receptor CD28, as well as the activity of *src* kinases and phospholipase C. Phosphoinositol 3-kinase (PI 3 K) and LFA-1 were not needed for ADAP regulation of HIV-1 LTR transcription. By contrast, SLP-76/ADAP regulation of viral cell-cell spread was reflected by a reduction in LFA-1-dependent DC-T or T-T cell conjugation by the absence of ADAP or expression of M12, as well as well as impaired formation of the VS between cells. Overall, our evidence shows that ADAP and its binding to SLP-76 regulates propagation of HIV-1 by two distinct coreceptors, and identifies the immune adaptor ADAP as a new possible target to control HIV-1 infection.

## Methods

### Cells

ADAP or M12 was subcloned into the retroviral vector pMXF5 containing IRES-GFP, and these plasmids were transfected in 293 T cells to prepare retroviral supernatants. Human C8166 and Jurkat T cells were transduced with these retroviral supernatants, and GFP^+^ cells were sorted by flow cytometry, which could stably express GFP vector or ADAP/GFP or M12/GFP. C8166 cells, Jurkat T cells, J14 (SLP-76 deficient) cells and JDAP (ADAP deficient) cells (a kind gift from Dr. Y. Huang, National Institutes of Health, Intramural Research Program/Department of Health and Human Services, Baltimore, Maryland, USA) were cultured in RPMI 1640 medium supplemented with 10% (v/v) fetal bovine serum (FBS), 100 U/ml penicillin, 100 μg/mL streptomycin at 37°C and 5% CO_2_. CD14^+^ monocytes were purified from human PBMCs (human peripheral blood mononuclear cells) using anti-CD14 antibodies-coated magnetic beads (BD Biosciences) and cultured with 50 ng/ml of granulocyte-macrophage colony stimulating factor (GM-CSF) (R&D) and IL-4 (R&D) for 6 days to generate immature DCs. Immature DCs were stimulated with LPS (10 ng/ml) for 48 h to generate mature DCs. Primary CD4^+^ T cells were purified from human PBMCs using anti-CD4 antibodies-coated magnetic beads (BD Biosciences) and activated with 5 μg/mL of phytohemagglutinin-P (PHA-P) (Sigma-Aldrich) for 72 h in the presence of 20 IU/mL of recombinant IL-2 (R&D).

### CA-p24 ELISA assay

To measure HIV-1 p24^Gag^ levels in the culture medium, culture supernatant was firstly heat inactivated at 56°C for 30 min in the presence of 0.05% Empigen-BB (Calbiochem, La Jolla, USA) and the CA-p24 concentration was determined by ELISA with D7320 (Biochrom, Berlin, Germany) as the capture antibody and alkaline phosphatase-conjugated anti-p24 monoclonal antibody (EH12-AP) as the detection antibody using a lumiphos plus system (Lumigen, Michigan, USA) in a LUMIstar Galaxy luminescence reader (BMG labtechnologies, Offenburg, Germany).

### HIV LTR driven transcription by luciferase assay

The pLTR-gag3-flag-luc plasmid contains the HIV-1 5’ LTR promoter region, the complete leader RNA, the N-terminal three Gag amino acids followed by the Flag peptide (amino acids DYKDDDDKD) and the firefly luciferase protein. The pLTR-gag3-flag-luc plasmid was transfected in Jurkat cells together with plasmids expressing ADAP/GFP, M12/GFP or GFP alone. Transfected cells were then seeded on to anti-CD3 and anti-CD28 or purified B7.1-Fc coated plate for 6 hrs. Cells were then harvested, lysed and measured for luciferase activity according to the protocol provided by Promega kits. Alternatively, transfected cells were treated with src kinase inhibitor PP2, PI3K inhibitor LY294002, PLCγ inhibitor U73122 or anti-LFA1 antibody (i.e. anti-CD18) over the incubation period.

### Knockdown of ADAP expression by siRNA

Specific siRNAs targeting human ADAP (5’-CCUGGUGAAUCUCUAGAAGTT-3’) or scrambled control siRNAs were transfected into human primary CD4^+^ cells using Lipofectamine 2000 (Invitrogen) as directed by the manufacturer. The levels of ADAP expression were examined by Western blotting at 48 h after transfection or by qRT-PCR at various time points.

### Immunoprecipitation, immunoblotting and EMSA assay

To check the activity of NF-κB, Jurkat and JDAP cells or C8166 cells over-expressing ADAP/GFP, M12/GFP and GFP control were stimulated with anti-CD3 (1 μg/ml) and anti-CD28 (2 μg/ml) antibodies for 30 min or indicated time. Nuclear extracts were prepared and incubated with biotin labelled NF-κB probes. Activated NF-κB formed a complex with NF-κB probes that could be detected according to Panomics’s protocol. Alternatively, cell lysates were prepared for immunoblotting with IκBα and actin to detect the degradation of IκBα.

### HIV-1 stocks and viral-like particles (VLPs)

CXCR4-tropic HIV-1 virus (pNL4.3) was generated by transfecting 293T cells as described below and infectivity determined by luciferase assay on HeLa tzmbl cells. HIV-1 viral stocks produced in C33A cells (24 wells plate) were produced by transfection of 1 μg of pLAI-R37. Pseudotyped single-cycle, luciferase reporter HIV stocks, HIV-Luc/NL4-3, were generated by calcium phosphate-mediated cotransfections of HEK293T cells with pLAI-Δenv-Luc, an env-deleted and nef-inactived HIV-1 proviral construct, and a construct expressing for HIV envelope protein (Env) of NL4-3 (X4-tropic) as described previously [12]. To produce HIV-1 VLPs, HIV-1-gag-GFP/NL4-3, were generated by cotransfection of HEK293T cells with a plasmid encoding HIV-gag-GFP and with an expression plasmid of NL4-3 Env. Supernatants that contain HIV-1 particles were harvested, filtered and titrated with p24^Gag^ capture ELISA.

### Virus infection and replication

Human primary CD4^+^ T cells knocking down of ADAP; C8166 cells and Jurkat cells stably overexpressing GFP or ADAP/GFP or M12/GFP; J14, JDAP or wild type Jurkat cells were respectively incubated with single-cycle HIV stocks (i.e. HIV-Luc/NL4-3 containing luciferase reporter, 1 ng or 5 ng of p24^Gag^) for 2 h at 37°C. After washing of excessive HIV-1 viruses, the above cells were incubated for further 3 days [12]. Alternatively, anti-LFA-1 or soluble ICAM-1-Fc was used to pre-treat T cells for 15 min and was kept in the culture medium during the incubation time. Cells were washed intensively post-infection and cell lysates were prepared to measure luciferase activity with a kit from Promega. Or, the amount of viruses was quantified by detecting HIV-1-gag mRNA levels with qRT-PCR using the forward primer (5’-GTGTGGAAAATCTCT AGCAGTGG-3’) and the reverse primer (5’-CGCTCTCGCACCCATCTC-3’). Actin was used as an internal reference.

### HIV-1 infection and transmission between T-T cells

T cells were infected with HIV-1 strain pNL4-3 by spinoculation and cells were cultured for 3 days before being used as HIV-1^+^ donor cells. 5 × 10^5^ ADAP/GFP or M12/GFP expressing target cells were mixed with 2.5 × 10^5^ HIV + donor T cells, incubated for 0, 6, 12 and 24 hr, and genomic DNA was extracted (Qiagen). Quantitative real-time PCR was performed to measure HIV *pol* DNA and the house-keeping gene *albumin* as described previously [10,13,14] The ratio of HIV *pol* DNA to *albumin* was determined as the HIV DNA copy number and the fold increase was calculated relative to the amount of HIV-1 DNA at the time point 0 hr as a measure of cell-cell spread.

### Conjugate or VS formation and immunostaining

For T-T conjugation, 5 × 10^5^ HIV^+^ donor cells were mixed with an equal number of target cells at 37°C on poly-L-lysine–treated coverslips for up to 1 hr as described previously [11]. Conjugates were fixed in 4% formaldehyde and permeabilized in 0.1% Triton X-100/5% FCS. Immunostaining of conjugates was performed using the following reagents: phalloidin-TRITC (Sigma-Aldrich), anti-Env mAb (mAb 50-69, donated by S. Zoller-Pazner and obtained from the CFAR, NIBSC, UK), rabbit antisera against HIV-1 Gag p17 and p24 (donated by G. Reid and obtained from the CFAR, NIBSC UK). To form DC-T conjugation, mature DCs (2 × 10^5^ cells) were pre-incubated with HIV-1-p24^Gag^-GFP/NL4-3 VLPs (20 ng of p24) at 37°C for 2 hr as previously described [12]. After extensive washes, these DCs were then incubated for 30 min at a ratio of 1:1 with Jurkat cells overexpressing ADAP/GFP or M12/GFP; J14 or JDAP; human primary CD4^+^ T cells knocking down of ADAP; and the control cells respectively. Conjugates were stained with anti-LFA-1 or anti-ADAP (BD Bioscience). Stained coverslips were mounted in Molwiol 4-88 (Calbiochem) or Prolong Gold antifade (Invitrogen), and analyzed using a confocal microscope linked to LSM 510™ software (Carl Zeiss MicroImaging, Inc.) or a Leica SP2.

### Statistics analysis

Data are presented as mean±SEM. A two-tailed Student’s t-test was used to compare two groups. ANOVA was used to analyze difference among three groups. For all test, a P value of 0.05 or less was considered statistically significant.

## Competing interests

We submit it as a regular article. All the authors concur with the submission. All the funding bodies and their recipients have been listed. All the data were generated by the current authors. This work has not been published elsewhere. We don’t have any financial or commercial conflicts of interests.

## Authors’ contributions

BW, LH, TA, EG, CJ, DL, RT, WG, HW performed experiments and statistical analysis. HW, CER, BW, CJ, JW participated in the design of the study. HW, CER, BW drafted the manuscript. TA, AL, CJ, JW, RZ provided viruses, cells, coordinate and helped to draft the manuscript. All authors read and approved the manuscript.

## Supplementary Material

Additional file 1: Figure S1 (A) After HIV-1 infection, Jurkat cells overexpressing GFP, ADAP/GFP or M12/GFP expressed surface CD4 and CXCR4 at the same levels. (B) C8166 T cells were stably transduced with GFP, ADAP/GFP or M12/GFP. The transduced efficiency reached over 95% according to the percent of GFP + cells by flow cytometry (left panel), and the expression levels of ADAP or M12 were assessed by immunoblotting (middle panel). These cells were infected with low or high doses (equivalent to 1.5 or 15 ng p24^Gag^, respectively) of HIV-1, and supernatants were collected at various times post infection to check the presence of p24^Gag^ by ELISA (right panel). Two independent experiments were performed and the representative data were collected from triplicate samples with error bars. (C) ADAP or M12 expression in C8166 cells did not affect the surface expression levels of CD4, CXCR4, CD3, CD28, β1 integrin or ICAM-1 as determined by flow cytometry. (D) Overexpression of ADAP or M12 in C8166 cells did not significantly alter cell proliferative capacity. (E) Knockdown of ADAP in human primary CD4^+^ T cells did not alter the surface expression levels of CD4, CXCR4, CD3, CD28, β1 or β2 integrins and ICAM-1. Click here for file

Additional file 2: Figure S2 The reporter plasmid pLTR-gag3-flag-luc contains the HIV-1 5’ LTR promoter region, three amino acids of Gag, the Flag tag, followed by the firefly luciferase open reading frame. (A) ADAP was cotransfected into Jurkat cells with the report plasmids expressing wild type HIV-1 LTR or the mutant LTR which lost NFB binding sites. The cells were then stimulated with anti-CD3/CD28 for 6 hrs to measure the luciferase readings. (B) Src kinase and PLCγ, but not PI3K, is essential for anti-CD3/CD28-induced HIV-1 transcription. Jurkat cells expressing GFP or ADAP/GFP were treated with specific inhibitors or anti-CD18, followed by a measurement of HIV-1 LTR transcription. Three independent experiments were performed and the representative data were collected from triplicate samples with error bars (* represents p = <0.05, ** represents p = <0.001). Click here for file

Additional file 3: Figure S3 (A) The surface expression levels of β2 integrin (i.e. CD18) on Jurkat and J14 cells were determined by flow cytometry. (B) Jurkat and JDAP cells were stimulated with plate-coated anti-CD3 and ICAM-1 (P = 0.0001). F-actin was stained with Phalloidin-TRITC to observe cell spreading. Click here for file
