# Supplementary figures and images for: Immune adaptor ADAP in T cells regulates HIV-1 transcription and cell-cell viral spread via different co-receptors
Source: Retrovirology. 2013 Sep 18;10:101. doi: 10.1186/1742-4690-10-101 (PMC3851709; doi:10.1186/1742-4690-10-101)

Fig. S1

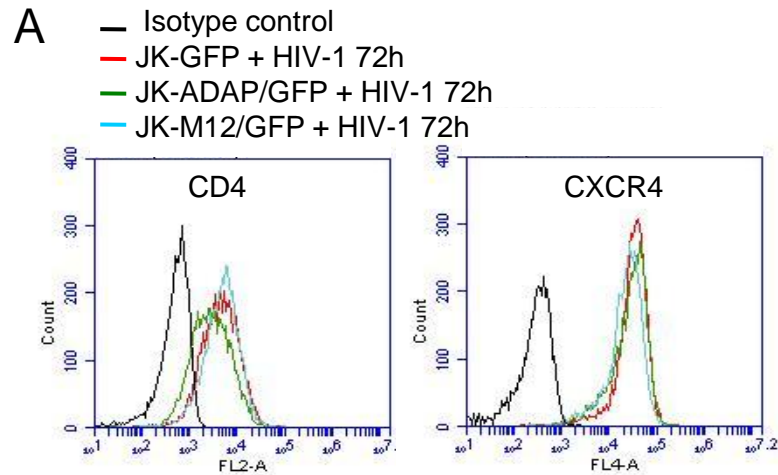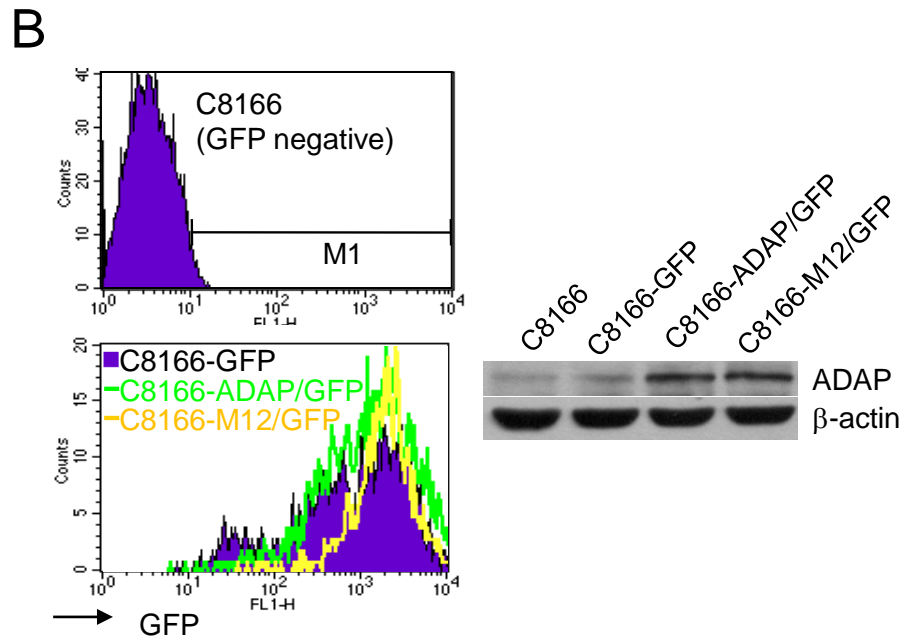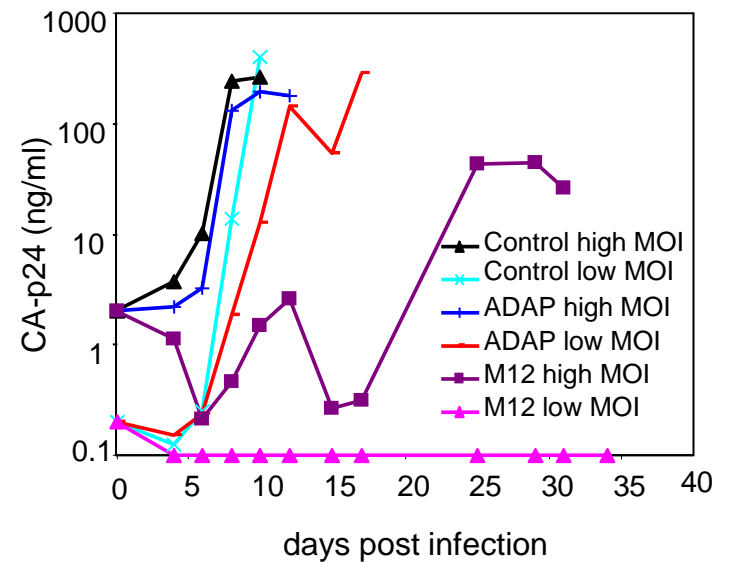

Fig. S1

C

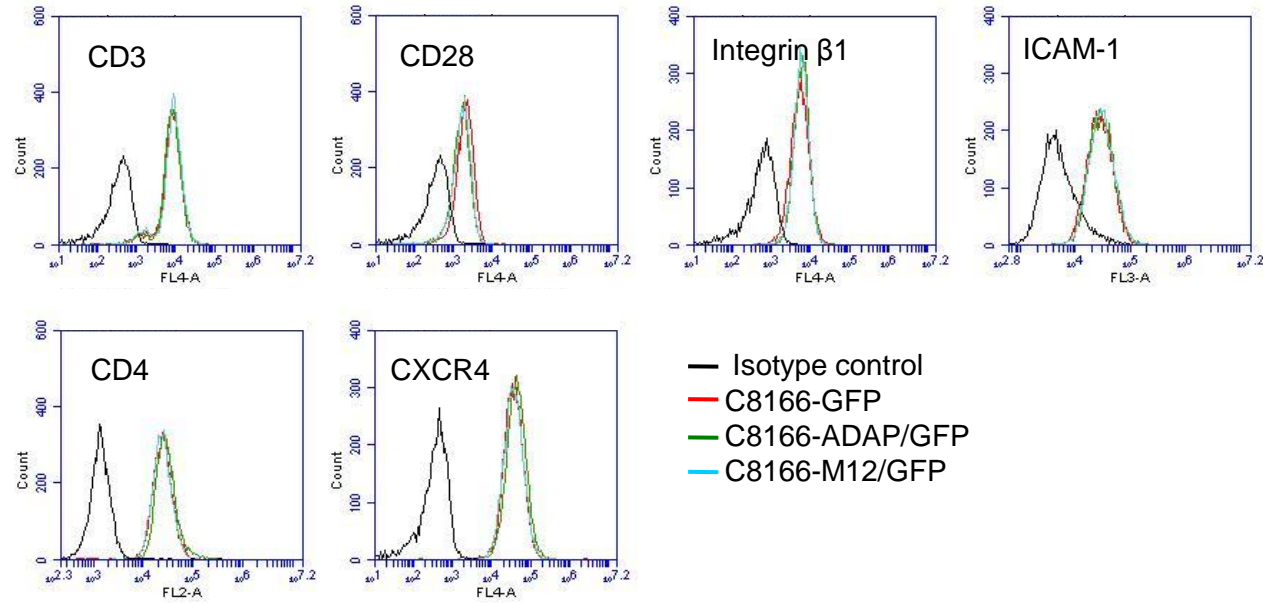

D

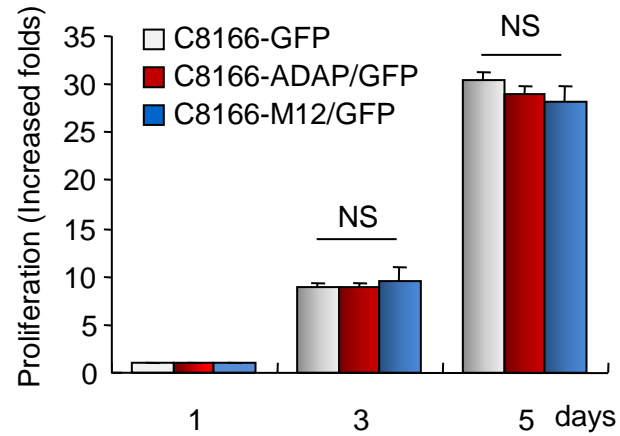

Fig. S1

E

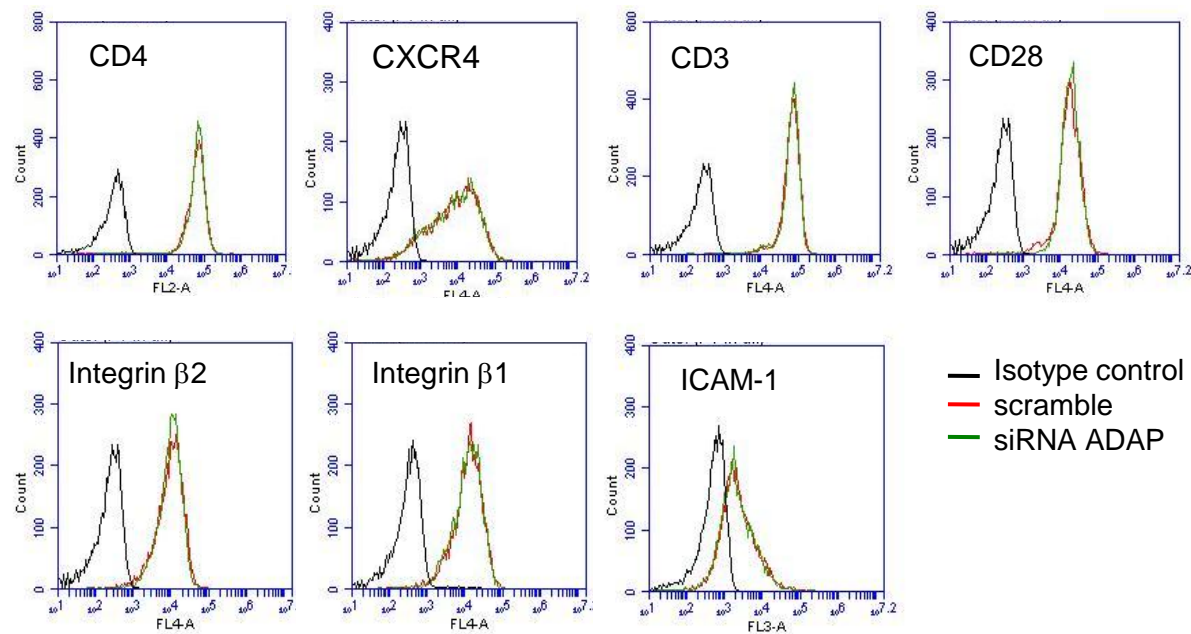

Supplement: Additional file 1: Figure S1 — (A) After HIV-1 infection, Jurkat cells overexpressing GFP, ADAP/GFP or M12/GFP expressed surface CD4 and CXCR4 at the same levels. (B) C8166 T cells were stably transduced with GFP, ADAP/GFP or M12/GFP. The transduced efficiency reached over 95% according to the percent of GFP + cells by flow cytometry (left panel), and the expression levels of ADAP or M12 were assessed by immunoblotting (middle panel). These cells were infected with low or high doses (equivalent to 1.5 or 15 ng p24Gag, respectively) of HIV-1, and supernatants were collected at various times post infection to check the presence of p24Gag by ELISA (right panel). Two independent experiments were performed and the representative data were collected from triplicate samples with error bars. (C) ADAP or M12 expression in C8166 cells did not affect the surface expression levels of CD4, CXCR4, CD3, CD28, β1 integrin or ICAM-1 as determined by flow cytometry. (D) Overexpression of ADAP or M12 in C8166 cells did not significantly alter cell proliferative capacity. (E) Knockdown of ADAP in human primary CD4+ T cells did not alter the surface expression levels of CD4, CXCR4, CD3, CD28, β1 or β2 integrins and ICAM-1. [file 1742-4690-10-101-S1.pdf]

Fig. S2

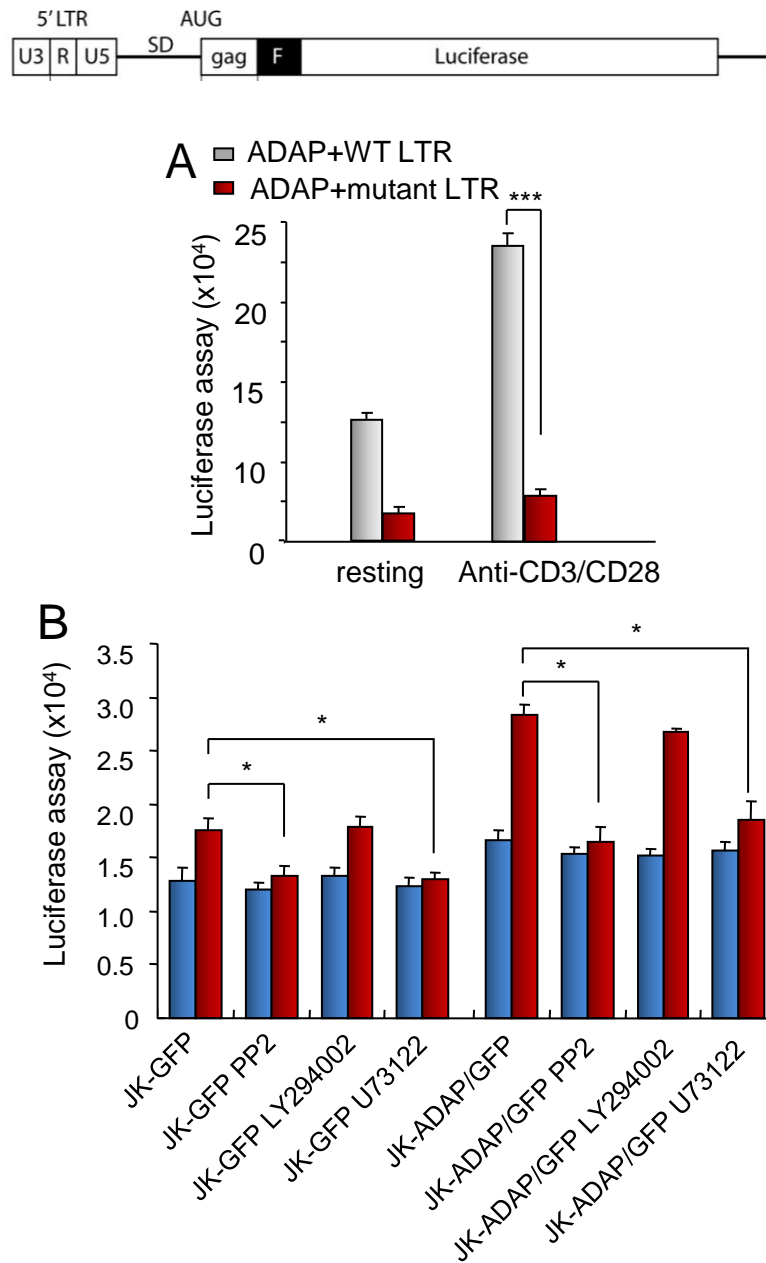

Supplement: Additional file 2: Figure S2 — The reporter plasmid pLTR-gag3-flag-luc contains the HIV-1 5’ LTR promoter region, three amino acids of Gag, the Flag tag, followed by the firefly luciferase open reading frame. (A) ADAP was cotransfected into Jurkat cells with the report plasmids expressing wild type HIV-1 LTR or the mutant LTR which lost NFB binding sites. The cells were then stimulated with anti-CD3/CD28 for 6 hrs to measure the luciferase readings. (B) Src kinase and PLCγ, but not PI3K, is essential for anti-CD3/CD28-induced HIV-1 transcription. Jurkat cells expressing GFP or ADAP/GFP were treated with specific inhibitors or anti-CD18, followed by a measurement of HIV-1 LTR transcription. Three independent experiments were performed and the representative data were collected from triplicate samples with error bars (* represents p = <0.05, ** represents p = <0.001). [file 1742-4690-10-101-S2.pdf]

Fig. S3

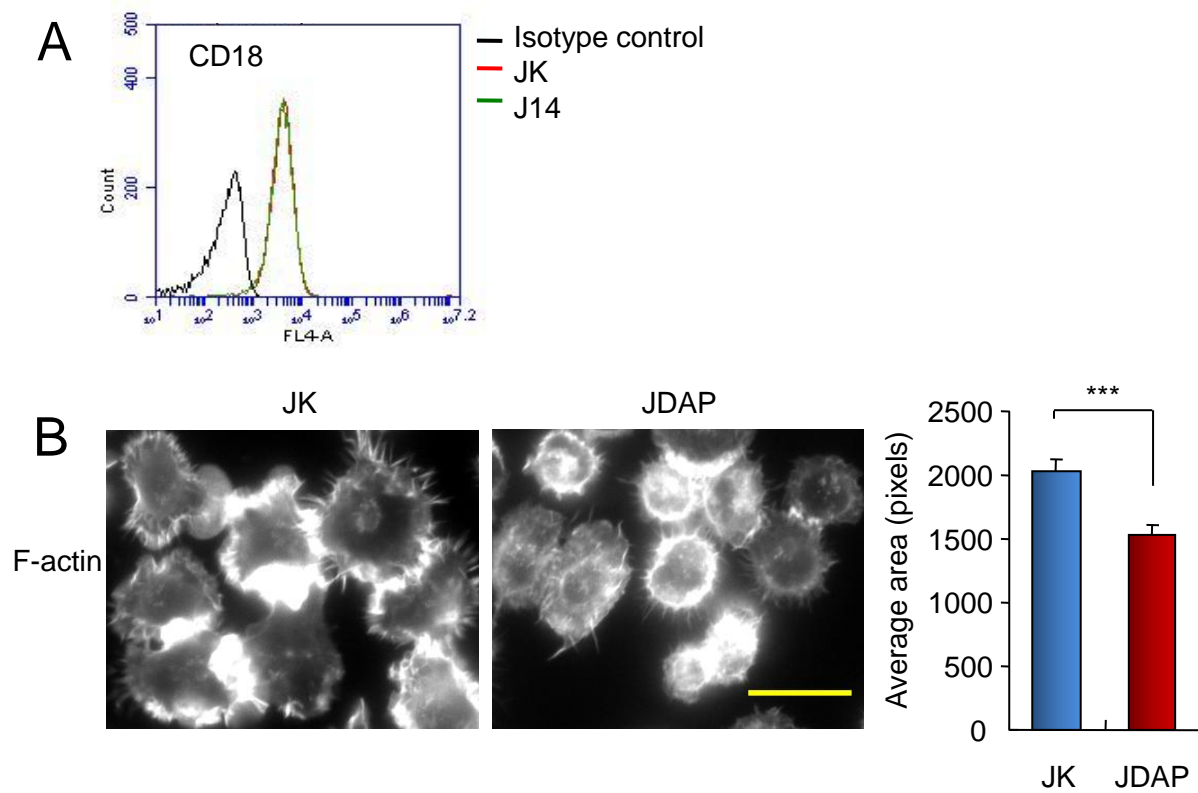

Supplement: Additional file 3: Figure S3 — (A) The surface expression levels of β2 integrin (i.e. CD18) on Jurkat and J14 cells were determined by flow cytometry. (B) Jurkat and JDAP cells were stimulated with plate-coated anti-CD3 and ICAM-1 (P = 0.0001). F-actin was stained with Phalloidin-TRITC to observe cell spreading. [file 1742-4690-10-101-S3.pdf]
